# Psychological Distress, Symptom Burden and Quality of Life in Patients with Mycosis Fungoides and Sézary Syndrome

**DOI:** 10.2340/actadv.v106.adv-2026-0463

**Published:** 2026-07-15

**Authors:** Rudy Bittar, Karolina Wojewoda, Martin Gillstedt, Catharina Lewerin, Mikael Alsterholm, Amra Osmancevic

**Affiliations:** 1 Department of Dermatology and Venereology, Institute of Clinical Sciences, Sahlgrenska Academy, University of Gothenburg, Gothenburg, Sweden; 2 Department of Dermatology and Venereology, Sahlgrenska University Hospital, Gothenburg, Region Västra Götaland, Sweden; 3 Section of Haematology and Coagulation, Department of Internal Medicine, Institute of Medicine, Sahlgrenska Academy, University of Gothenburg, Gothenburg, Sweden

**Keywords:** mycosis fungoides, Sézary syndrome, quality of Life, depression, anxiety

## Abstract

Mycosis fungoides (MF) and Sézary syndrome (SS) are rare cutaneous lymphomas with limited data on psychological burden. This observational, cross-sectional questionnaire study assessed symptom burden, psychological distress and quality of life (QoL) in patients with MF and SS in Western Sweden and compared outcomes with psoriasis. Sixty patients were included, 80% with early-stage disease. Most reported minimal depression, anxiety and QoL impairment. Moderate-to-severe depression occurred in 36% of advanced disease cases and 16% of early disease cases and moderate-to-severe QoL impairment in 33% and 17%, respectively. Pruritus correlated strongly with impaired QoL (ρ=0.67, *p*<0.001). Fatigue correlated with depression (Montgomery–Åsberg Depression Rating Scale – Self-report (MADRS-S): ρ=0.60, Hospital Anxiety and Depression Scale-Depression subscale: ρ=0.63), anxiety (ρ=0.49) and QoL impairment (ρ=0.53), all *p*<0.001. Compared with psoriasis, patients reported lower pain and QoL impairment. Overall, psychological burden was limited but increased with advanced disease and fatigue severity.

**SIGNIFICANCE**

This study shows that most people living with mycosis fungoides or Sézary syndrome experience limited psychological distress and impact on daily life. Those with more advanced disease face a greater psychological burden. It also highlights that fatigue and itching are key symptoms linked to reduced quality of life. Compared with psoriasis, patients reported more fatigue but less pain. These findings underline the importance of routine inquiry about psychological health and symptom burden in clinical care, particularly in advanced disease, to ensure timely interventions.

Primary cutaneous lymphomas (PCL) are rare non-Hodgkin lymphomas confined to the skin at diagnosis (1). They are classified into cutaneous T-cell lymphomas (CTCL) and cutaneous B-cell lymphomas, with CTCL representing approximately 75% of cases. Mycosis fungoides (MF) is the most common CTCL subtype, accounting for about 60% of cases, and typically follows an indolent course characterized by patches, plaques and sometimes tumours (2). In contrast, Sézary syndrome (SS) is a rare but more aggressive leukemic CTCL variant, comprising roughly 2.5% of cases. It is defined by erythroderma with peripheral blood and lymph node involvement (3). The prognosis varies considerably by stage, with early MF (stages IA–IIA) demonstrating a favourable 5-year survival rate exceeding 90%. In contrast, advanced disease (stages IIB–IV) shows a substantially lower 5-year survival of approximately 40% (4).

Previous studies have documented significant quality of life (QoL) impairment in MF/SS patients, with greater burden observed in advanced stages (5–11). However, the literature reveals considerable hetero-geneity in reported outcomes, attributable to differences in study populations, disease stage distributions, assessment instruments and clinical contexts (5–17). Pruritus has consistently emerged as one of the most prevalent and bothersome symptoms, affecting up to 88–94% of patients and strongly correlating with reduced QoL (18). Fatigue, though less extensively studied, has also been identified as a common and burdensome symptom in CTCL populations.

Comparative studies examining QoL between MF/SS and other chronic inflammatory skin diseases are limited. Psoriasis shares certain clinical features with early MF, including erythematous plaques and potential for significant QoL impairment (19). In addition, symptoms such as fatigue are clinically relevant in both inflammatory and lymphoproliferative disease and are not fully captured by conventional measures of skin disease severity. Understanding how symptom profiles and QoL burden differ between these conditions may provide insights into a more targeted approach and management of these diseases, aiming to improve overall patient care.

Despite growing recognition of the importance of patient-reported outcomes in MF/SS, several knowledge gaps persist. First, psychological distress, QoL and symptom burden are still unknown in MF/SS patients in Western Sweden. Second, the relative contribution of specific symptoms, such as fatigue and pruritus, to overall psychological distress and QoL impairment remains incompletely characterized. Third, direct comparisons with other chronic skin diseases using standardized instruments are scarce.

The aim of this cross-sectional study was to assess symptom burden, psychological distress, and QoL in patients with MF and SS in Western Sweden using validated instruments. Patient-reported outcomes were also compared to psoriasis patients to identify disease-specific patterns of impairment.

## MATERIALS AND METHODS

### Study design

This cross-sectional observational study included patients with MF or SS at various stages of their disease trajectory, identified through the PCL register of Western Sweden, as previously described by Wojewoda et al. (20, 21). Medical records for all patients included in the PCL register from 1 January 2005, to 4 February 2025 were reviewed. Exclusion criteria were deceased, lack of written consent for research participation, age under 18 years, PCL diagnosis other than MF/SS or unverified MF/SS diagnosis.

### Outcome measurements

Outcomes were symptom burden, psychological distress and health-related QoL, reported by patients with MF and SS, with the following validated questionnaires in Swedish: Visual Analogue Scale (VAS) for pruritus, pain and fatigue (22), Montgomery–Åsberg Depression Rating Scale – Self-report (MADRS-S) (23), Hospital Anxiety and Depression Scale (HADS) (24) and Dermatology Life Quality Index (DLQI) (25). Data on gender, age at disease onset, clinical stage, body mass index (BMI) and the presence of cardiometabolic comorbidities were obtained from medical records when missing from the PCL register. Cardiometabolic diseases were defined as hypertension, hyperlipidaemia, type II diabetes mellitus and/or a history of myocardial infarction.

VAS scores for pain and fatigue, HADS and DLQI were compared with a Swedish cohort of 139 patients with psoriasis, GÖTHA. Participants in the comparison group were between 18 and 80 years of age and had a confirmed diagnosis of psoriasis. Exclusion criteria for this cohort were inability to complete questionnaires in Swedish, inability to follow the study protocol and a diagnosis of psoriatic arthritis or other rheumatic disease. Comparative analyses of patient-reported outcomes between the MF/SS cohort and the psoriasis cohort served as the secondary outcome.

### VAS

The severity of pruritus, pain and fatigue experienced in the past 24 h was assessed using separate VAS, 1 for each item. Each scale ranged from 0 to 10, where 0 indicated the absence of the symptom and 10 the most severe intensity (22).

### MADRS-S

To assess depression, MADRS-S was used, which consists of nine items related to patients’ mood, feelings of unease, sleep, appetite, ability to concentrate, initiative, emotional involvement, pessimism and zest for life (23). Each item is scored 0–6, with total scores of 0–12 indicating no or minimal depression, 13–19 mild depression and ≥20 moderate to severe depression (26).

### HADS

Anxiety and depression were assessed using the 14-item HADS, with 7 items each for anxiety (HADS-A) and depression (HADS-D) (27). Items are scored 0–3, with subscale scores of 0–7 considered normal, 8–10 borderline and ≥11 indicative of clinically relevant anxiety or depression.

### DLQI

The 10-item DLQI was used to assess the impact of skin disease on QoL (25). Each item is scored 0–3, for a total of 0–30. Scores of 0–1 indicate no impact, 2–5 small, 6–10 moderate and ≥11 very large to extremely large impact on QoL (28).

### Statistical analysis

Data were analyzed using R version 3.5.3 (The R Foundation for Statistical Computing, Vienna, Austria). All tests were 2-sided, with *p*<0.05 considered statistically significant. Sum scores were calculated per instrument manuals. Continuous data are presented as means and medians, categorical data as frequencies and percentages. Group differences were assessed using Fisher exact and Wilcoxon rank-sum tests. Associations between symptom severity and psychological distress/QoL impairments were analysed using Spearman rank correlations. The Benjamini–Yekutieli procedure was used to adjust for multiple hypothesis tests.

## RESULTS

**Fig. 1.**
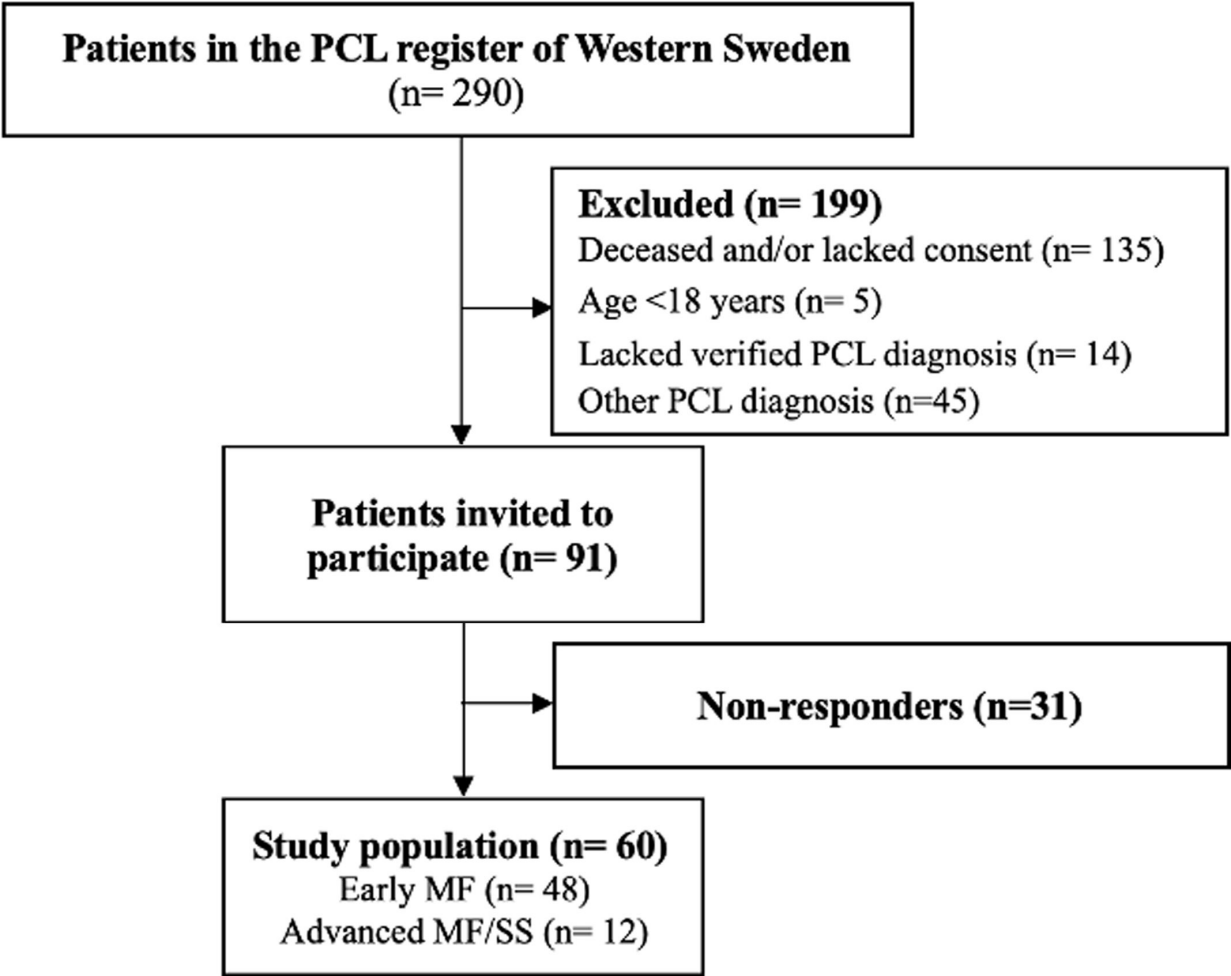
Flowchart illustrating the selection procedure of the study population. PCL: primary cutaneous lymphoma; MF :mycosis fungoides; SS: Sézary syndrome.

Of the 91 patients with MF/SS invited to participate, 60 responded, resulting in a response rate of 66% (Fig. 1). The cohort was subdivided into early MF (*n*=48) and advanced MF/SS (*n*=12), of whom 7 had SS. Overall, 63% of the cohort were male, resulting in male:female distribution of 1.7 : 1. The age at disease onset was significantly lower among patients with early MF (median: 48.0 years) compared to those with advanced MF/SS (median: 67.6 years, *p*=0.01) (Table I). Similarly, the age at diagnosis was significantly lower in patients with early MF (median: 55.7 years) compared to those with advanced MF/SS (median: 67.2 years, *p*=0.04).

**Table I.** Demographic and clinical characteristics of the cohort

Variable	Total (*n*=60)	Early MF (*n*=48)	Advanced MF/SS (*n*=12)	*p*-value
**Stage,** ***n*** **(%)**		IA 34 (71%) IB 11 (23%) IIA 3 (6%)	IIB 5 (42%) IVA1 5 (42%) IVA2 1 (8%) NA 1 (8%)	
**Sex,** ***n*** **(%)**				
Male	38 (63%)	31 (65%)	7 (58%)	*p*=0.74^a^
Female	22 (37%)	17 (35%)	5 (42%)	
**Age at disease onset (years)**				
Median (range)	53.0 (9.1–85.2)*	48.0 (9.1–85.2)	67.6 (31.4–79.5)*	***p*** **=0.01** ^b^
Mean±SD	50.2±20.5*	47.0±20.6	64.1±13.6*	
**Age at diagnosis (years)**				
Median (range)	57.4 (20.0–85.6)	55.7 (20.0–85.6)	67.2 (40.7–82.2)	***p*** **=0.04** ^b^
Mean±SD	56.3±17.0	54.2±17.0	64.8±14.1	
**Body mass index (kg/m²)**				
Median (range)	26.0 (18.2–37.2)**	25.8 (18.2–37.2)**	26.6 (20.3–34.0)	*p*=0.68^b^
Mean±SD	26.3±4.4 **	26.2±4.6**	26.5±3.8	
**Cardiometabolic disease,** ***n*** **(%)**				
Yes	25 (42%)	18 (38%)	7 (58%)	*p*=0.21^a^
No	35 (58%)	30 (63%)	5 (42%)	
**Depression diagnosis,** ***n*** **(%)**				
Yes	10 (17%)	7 (15%)	3 (25%)	*p*=0.40^a^
No	50 (83%)	41 (85%)	9 (75%)	

^a^Fisher exact test used to calculate p-value, Early MF vs Advanced MF/SS. ^b^Wilcoxon rank-sum test used to calculate *p*-value Early MF vs Advanced MF/SS. Bold font indicates statistically significant *p*-value.

*data missing for 1 patient; **, data missing for 10 patients.

MF: Mycosis fungoides; NA: not known; SD: standard deviation; SS: Sézary syndrome.

### Questionnaire outcomes in the MF/SS cohort

Most patients with MF/SS reported no to minimal depression (MADRS-S, HADS-D) and anxiety (HADS-A), and low disease-related QoL impact (DLQI) (Table II). Median scores were 7.5 (interquartile range [IQR]: 0.5–14) for MADRS-S, 2 (IQR: 1–6) for HADS-D, 5 (IQR: 2–7) for HADS-A and 1 (IQR: 0.5–3) for DLQI, reflecting generally low levels of depression, anxiety and impact on QoL (S1). Analysis of VAS scores for pruritus, pain and fatigue revealed that fatigue was the most prominent symptom in this patient group, with a median VAS score of 3 (IQR: 0–5), followed by pruritus with a median VAS score of 2 (IQR: 0–4)(Table II).

**Table II.** Questionnaire outcomes assessing psychological distress, quality of life and symptom burden

Questionnaire	Total (*n*=60)	Early MF (*n*=48)	Advanced MF/SS (*n*=12)	*p*-value^a^
**MADRS-S,** ***n*** **(% of valid responses)**				
No to minimal depression (0–12 p) Mild depression (13–19 p) Moderate to severe depression (≥20 p) Invalid	37 (69%) 7 (13%) 10 (19%) 6	30 (70%) 6 (14%) 7 (16%) 5	7 (64%) 0 4 (36%) 1	*p*=0.68
**HADS-A,** ***n*** **(% of valid responses)**				
Normal (0–7p) Borderline (8–10p) Presence of anxiety (≥11p) Invalid	45 (76%) 8 (14%) 6 (10%) 1	37 (79%) 6 (13%) 4 (9%) 1	8 (67%) 2 (17%) 2 (17%) 0	*p*=0.71
**HADS-D,** ***n*** **(% of valid responses)**				
Normal (0–7p) Borderline (8–10p) Presence of depression (≥11p) Invalid	48 (81%) 4 (7%) 7 (12 %) 1	40 (85%) 2 (4%) 5 (11%) 1	8 (67%) 2 (17%) 2 (17%) 0	*p*=0.52
**DLQI,** ***n*** **(% of valid responses)**				
No impact (0–1p) Small impact (2–5p) Moderate impact (6–10p) Large impact (≥11p) Invalid	34 (58%) 13 (22%) 8 (14%) 4 (7%) 1	27 (57%) 12 (26%) 5 (11%) 3 (6%) 1	7 (58%) 1 (8%) 3 (25%) 1 (8%) 0	*p*=0.80
**VAS pruritus**				
Median (IQR) Mean (range)	2 (0–4) 2.4 (0–9)	2 (0–4.25) 2.67 (0–9)	0 (0–2.25) 1.5 (0–7)	*p*=0.15
**VAS pain**				
Median (IQR) Mean (range)	0 (0–1) 1.25 (0–8)	0 (0–1.25) 1.35 (0–8)	0 (0–0) 0.83 (0–8)	*p*=0.24
**VAS fatigue**				
Median (IQR) Mean (range)	3 (0–5)* 3.3 (0–10)*	3 (0–5)* 3.18 (0–9)*	3.5 (0.75–6.25) 3.75 (0–10)	*p*=0.56

Percentages are calculated based on valid responses only. Missing data is considered invalid and is therefore excluded from the denominator. ^a^Wilcoxon rank-sum test used to calculate *p*-value for raw data, Early MF vs Advanced MF/SS.

*data missing for 3 patients.

DLQI: Dermatology Life Quality Index; HADS-A: Hospital Anxiety and Depression Scale – Anxiety Subscale; HADS-D: Hospital Anxiety and Depression Scale – Depression Subscale; IQR: interquartile range; MADRS-S: Montgomery-Åsberg Depression Rating Scale – Self-Reported; MF:Mycosis fungoides; SS: Sézary syndrome; VAS: Visual Analogue Scale.

**Fig. 2.**
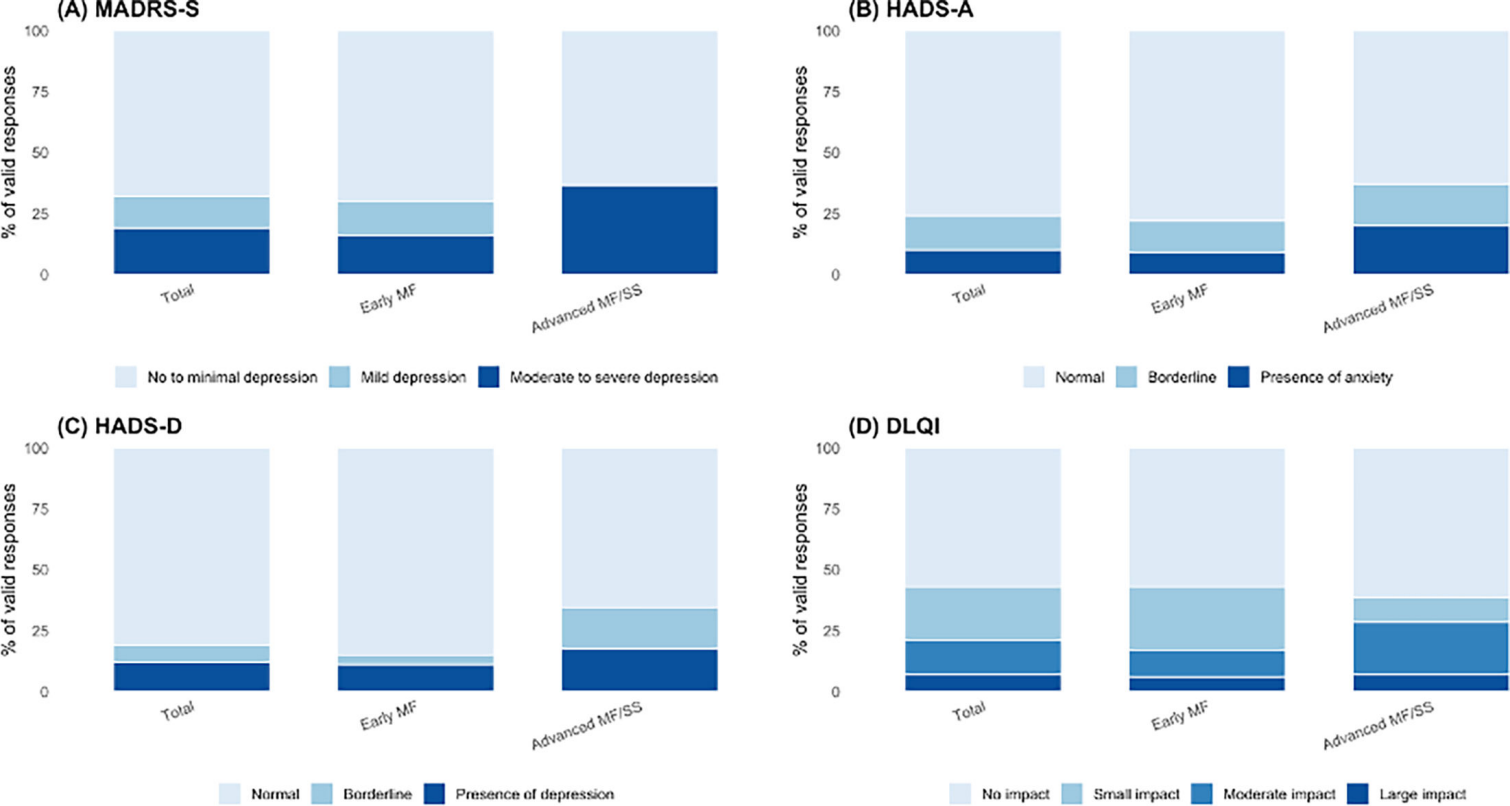
Stacked bar plots illustrating the distribution of symptom severity for anxiety, depression and quality of life impairment across the total MF/SS cohort and disease subgroups (Early MF vs Advanced MF/SS). Severity was categorized using validated cut-offs for MADRS-S, HADS-D, HADS-A and DLQI. Invalid data were excluded. MF: mycosis fungoides; SS: Sézary syndrome; MADRS-S: Montgomery–Åsberg Depression Rating Scale – Self-reported; HADS-A: Hospital Anxiety and Depression Scale – Anxiety subscale; HADS-D: Hospital Anxiety and Depression Scale – Depression subscale; DLQI: Dermatology Life Quality Index.

Stratification by disease stage revealed no statistically significant differences between early and advanced MF/SS (Fig. 2). Moderate-to-severe depression (MADRS-S) was observed in 36% (*n*=4) of advanced and 16% (*n*=7) of early MF patients (*p*=0.68), while moderate-to-severe QoL impairment (DLQI≥6) was reported in 33% (*n*=4) and 17% (*n*=8), respectively (*p*=0.8). No significant differences were observed in symptom burden (VAS pruritus, VAS pain and VAS fatigue), although median VAS pruritus was 0 (IQR 0–2.25) in advanced MF/SS and 2 (IQR 0–4.25) in early MF (*p*=0.15).

### Correlations between symptom burden and questionnaire outcomes in the MF/SS cohort

Correlation analyses revealed several statistically significant associations between symptom burden and questionnaire outcomes. VAS pruritus showed a strong positive correlation with DLQI (ρ=0.67, *p*<0.001) (Table III). By comparison, VAS fatigue correlated moderately to strongly with all psychological and QoL measures, including MADRS-S (ρ=0.60), HADS-A (ρ=0.49), HADS-D (ρ=0.63), and DLQI (ρ=0.53), with all associations showing statistical significance (*p*<0.001).

**Table III.** Correlations between symptom burden assessed by VAS and questionnaire-based measures of anxiety, depression, and quality of life in Mycosis fungoides and Sézary syndrome

**VAS Questionnaire**	**MADRS-S**			**HADS-A**			**HADS-D**			**DLQI**		
	ρ^a^	***p*** **-value** ^**b**^		ρ^a^	***p*** **-value** ^b^		ρ^a^	***p*****-v**a**lue**^b^		ρ^a^	***p*** **-value** ^b^	
VAS pruritus	0.32	*p*=0.081		0.21	*p*=0.39		0.16	*p*=0.74		0.67	***p*** **<0.001**	
VAS pain	0.41	***p*** **=0.012**		0.26	*p*=0.19		0.26	*p*=0.19		0.53	***p*** **<0.001**	
VAS fatigue	0.60	***p*** **<0.001**		0.49	***p*** **<0.001**		0.63	***p*** **<0.001**		0.53	***p*** **<0.001**	

^a^Correlations calculated according to Spearman rank correlation test. ^b^The Benjamini–Yekutieli procedure was used to adjust for multiple hypothesis tests. *p*-value presented are adjusted for multiple testing. Bold font indicates statistically significant *p*-value.

DLQI: Dermatology Life Quality Index; HADS-A: Hospital Anxiety and Depression Scale – Anxiety Subscale; HADS-D: Hospital Anxiety and Depression Scale – Depression Subscale; MADRS-S: Montgomery-Åsberg Depression Rating Scale – Self-Reported; VAS: Visual Analogue Scale; ρ: Spearman's rho.

### Comparison of the questionnaire outcomes in the MF/SS cohort compared to the psoriasis cohort

The psoriasis cohort included 139 patients, of whom 76 (55%) were male, resulting in a sex distribution comparable to that of the MF/SS cohort (S2). At the time of assessment, psoriasis severity was generally mild, with a median PASI of 3.6 (IQR 1.4–5.4, range 0–12). Similarly, disease burden in the MF/SS cohort was limited, with 80% (*n*=48) of patients presenting with early-stage disease. Thus, both cohorts predominantly consisted of patients with mild or early disease, reducing the likelihood that observed differences in questionnaire outcomes were driven by differences in disease severity. Both cohorts had a median BMI in the overweight range (MF/SS: 26 kg/m², psoriasis: 27 kg/m²). Cardiometabolic comorbidities were more prevalent in the psoriasis cohort (78%, *n*=91) than in MF/SS (42%, *n*=25), *p*<0.001. Most patients in both cohorts had no documented history of depression (MF/SS: 83%, *n*=50; psoriasis: 74%, *n*=100).

Comparison of questionnaire outcomes showed that moderate-to-large QoL impairment was reported by 37% (*n*=51) of psoriasis patients compared to 21% (*n*=12) in the MF/SS cohort, with median DLQI significantly higher in psoriasis (3, IQR 1–8.25 vs 1, IQR 0.5–3, *p*=0.001) (Table IV). VAS pain scores were also significantly higher in psoriasis (0.8, IQR 0.1–1.9 vs 0, IQR 0–1, *p*<0.001). VAS fatigue scores were 3 (IQR 0–5) in the MF/SS cohort and 1.5 (IQR 0.3–3.7) in the psoriasis cohort (*p*=0.49).

**Table IV.** Descriptive statistics comparing questionnaire outcomes between the MF/SS and psoriasis cohorts

Questionnaire	MF and SS (*n*=60)	Psoriasis (*n*=139)	*p*-value^**a**^
**HADS-A** Median (IQR) Mean (range)	5 (2–7)* 5.12 (0–17)*	5 (2–9)** 5.95 (0–19)**	*p*=0.29
**HADS- D** Median (IQR) Mean (range)	2 (1–6)* 3.92 (0–16)*	2 (1–6)** 3.55 (0–14)**	*p*=0.58
**DLQI** Median (IQR) Mean (range)	1 (0.5–3)* 2.8 (0–13)*	3 (1–8.25)*** 5.1 (0–22)***	***p*** **=0.001**
**VAS pain** Median (IQR) Mean (range)	0 (0–1) 1.25 (0–8)	0.8 (0.1–1.9)**** 1.58 (0–9.4)****	***p*** **<0.001**
**VAS fatigue** Median (IQR) Mean (range)	3 (0–5) 3.3 (0–10)	1.5 (0.3–3.7) 2.4 (0–9.9)	*p*=0.49

^a^Wilcoxon rank-sum test used to calculate *p*-values for MF and SS vs psoriasis. Bold font indicates statistically significant *p*-values.

*data missing for 1 patient; **data missing for 2 patients; ***data missing for 3 patients; ****data missing for 4 patients.

DLQI: Dermatology Life Quality Index; HADS-A: Hospital Anxiety and Depression Scale – Anxiety Subscale; HADS-D: Hospital Anxiety and Depression Scale – Depression Subscale; IQR: interquartile range; MF: Mycosis fungoides; SS: Sézary syndrome; VAS: Visual Analogue Scale.

## DISCUSSION

This cross-sectional study examined psychological distress, symptom burden, and the impact of disease on QoL in patients with MF/SS and compared the findings with a psoriasis cohort. Overall, most MF/SS patients reported minimal to no depression, anxiety or QoL impairments. Psychological distress and QoL impairments were more pronounced in advanced stages. Fatigue emerged as the most prominent symptom and correlated moderately to strongly with both psychological distress and impaired QoL. Pruritus was less prominent but strongly associated with poorer QoL. Compared with the psoriasis cohort, patients with psoriasis reported greater impairment in QoL and higher pain scores than MF/SS patients. Together, these findings highlight important differences in symptom profiles between MF/SS and psoriasis.

The overall psychological distress observed in this MF/SS cohort was low and consistent with prior literature (12, 14, 16, 29–32). However, when compared with a cohort of patients with CTCL recruited during routine clinical care at the Department of Dermatology, Karolinska University Hospital, Stockholm, and assessed with the same depression instrument (MADRS-S), a higher proportion of patients in our study reported mild-to-severe depressive symptoms (32% vs 13%) (14). This difference cannot be explained solely by stage distribution, as depressive symptoms were also reported in approximately 30% of early-stage patients. One possible explanation may relate to differences in data collection periods, as seasonal variation in daylight exposure has been suggested to influence mood (33). In contrast, anxiety and depression outcomes measured by HADS were largely consistent with other studies restricted to early MF (12, 32). Although we did not observe statistically significant differences between early and advanced MF/SS, the proportion of patients reporting depression or anxiety tended to be higher in advanced disease, in line with previous reports (31). A possible explanation for the lack of statistical significance is the low number of patients with advanced MF/SS in this cohort.

QoL impairment in MF/SS has been widely documented, with large variations in magnitude (5–17). Several methodological factors may account for this variability. First, the patient-reported outcome instruments used range from dermatology-specific questionnaires to cancer-specific or generic questionnaires. Dermatology-specific questionnaires (e.g. DLQI, Skindex-29, Skindex-16) use different domains, scoring systems and cut-offs and that complicates direct comparisons. Second, study populations differ substantially, from the broader CTCL spectrum to early-stage MF only. Even among CTCL studies, the proportion of early vs advanced disease varies (5, 6, 9–11, 13, 14, 16, 17, 29). In addition, the clinical context of investigation differs, with some studies assessing patients at diagnosis, others during ongoing therapy, and some during therapy switch, all of which may influence perceived QoL. Third, the healthcare setting and geographic context may play a role. The proportion of patients reporting low QoL impairment in our cohort is comparable to those reported in other European studies (9, 12, 14). Consistent with prior literature, we observed a trend toward greater QoL impairment in more advanced disease stages (5–11). This is clinically plausible, as advanced MF/SS is associated with greater skin involvement, tumour burden, systemic symptoms and more intensive treatment, which may impair QoL and well-being. However, differences were not statistically significant, likely due to early-stage predominance and few advanced cases, limiting statistical power.

Fatigue was the most prominent symptom in this cohort, followed by pruritus and, to a lesser extent, pain. Other studies have identified fatigue as one of the most common and burdensome symptoms in patients with CTCL (5, 34). The aetiology of fatigue in MF/SS is likely multifactorial, reflecting the combined effects of chronic inflammatory activity, malignancy-related systemic burden and treatment-related factors (35). Psychological distress may also be a contributing factor, as depression and anxiety can cause fatigue and insomnia. Notably, in our cohort, fatigue showed moderate to strong correlations with both anxiety, depression and impaired QoL. Concerning pruritus, our findings are broadly consistent with previous reports demonstrating its high prevalence and its significant impact on QoL (7, 10, 11). We did not observe higher pruritus levels in advanced disease, as has been described earlier. Instead, median VAS pruritus was lower in advanced stages. This finding may reflect effective, stage-adapted treatment resulting in well-controlled symptoms, as well as the low representation of patients with advanced MF/SS in our cohort. Nevertheless, pruritus showed a strong correlation with impaired QoL. According to Ottevanger et al., pruritus substantially affects sleep, daily functioning, mood and social interactions, thereby contributing to overall disease burden (18).

In comparison with the psoriasis cohort, patients with mild psoriasis in our study reported greater QoL impairment and higher pain levels than patients with MF/SS. Tamer et al. assessed QoL in 60 patients with psoriasis and 50 patients with MF and reported a median DLQI of 4 and 1, respectively, which is consistent with our results (36). The authors suggested that the greater QoL impairment in psoriasis may be explained by longer disease duration and higher age. These differences were not observed in our cohorts. One possible explanation for this is the predominance of early-stage MF in our study population. Early MF is often clinically indolent and stable, which may result in a lower perceived disease burden, while patients with advanced disease, who may experience greater impairment, were underrepresented. Although the psoriasis cohort had mild disease, psoriasis is associated with comorbid conditions that may negatively affect QoL, including musculoskeletal involvement, cardiometabolic disease and chronic pain (37). Consistent with this, the prevalence of cardiometabolic disease was significantly higher in the psoriasis cohort than in the MF/SS cohort. Nevertheless, cardiometabolic comorbidity is also relevant in MF/SS. Johnson et al. reported higher prevalence of diabetes mellitus and hypertension in patients with MF/SS compared with healthy controls (38), suggesting that cardiometabolic risk is also elevated in this patient group. This may be related to chronic immune dysregulation and the effects of systemic inflammation on endothelial function and metabolic homeostasis, as well as additional contributing factors such as reduced physical activity, treatment-related factors and psychological distress. Although these comorbidities appear less pronounced than in psoriasis cohorts, they remain clinically relevant in MF/SS and should be considered when interpreting patient-reported outcomes and long-term disease burden.

One strength of this study is the relatively high response rate (66%), supporting the representativeness and reliability of the data. Questionnaire distribution, collection and data handling were conducted by the same researcher, ensuring procedural consistency and reduced administrative variability. The use of well-validated instruments (MADRS-S, HADS, DLQI and VAS) strengthens the internal validity and enables comparison with prior literature. The cohort consisted predominantly of patients with early MF, resulting in underrepresentation of advanced disease, which likely limited detection of stage-related differences. The overall sample size was modest, further reducing statistical power and generalizability. The cross-sectional design captures patient experience at a single time point. Participants were in different phases of the disease trajectory, including newly diagnosed patients, those with long-standing disease and those undergoing treatment changes. These temporal and clinical variations may have influenced psychological adaptation, symptom perception and reported QoL. Information on treatment was not collected, limiting evaluation of its influence on symptom burden and patient-reported outcomes. Disease severity was not assessed using the modified Severity-Weighted Assessment Tool, and therefore, the relationship between skin disease burden and patient-reported outcomes could not be evaluated.

In conclusion, this study highlights the importance of incorporating symptom-specific and patient-reported outcome assessments into the routine management of MF/SS. Although overall psychological distress and QoL impairment were low in this cohort, the observed trends toward greater burden in advanced disease suggest that patients with advanced stages may benefit from closer psychological monitoring. Notably, fatigue emerged as the symptom most strongly associated with both psychological distress and reduced QoL and needs to be addressed in clinical practice. While pruritus remains a key dermatologic symptom and an important driver of QoL impairment, fatigue may represent a broader indicator of overall disease burden that is not captured by skin severity measures alone. Additionally, the strong association between pruritus and impaired QoL highlights the potential value of targeted symptom management as part of routine patient care. And finally, the differences observed between MF/SS and psoriasis emphasize that symptom profiles and their impact on daily functioning vary between chronic skin diseases, even when objective disease severity appears mild.

## Data Availability

The anonymized raw data supporting this article’s conclusions are available from the authors upon reasonable request. Requests should be directed to amra.osmancevic@vgregion.se.
