# Supplementary material for: Psychological Distress, Symptom Burden and Quality of Life in Patients with Mycosis Fungoides and Sézary Syndrome
Source: Acta Derm Venereol. 2026 Jul 15;106:0463. doi: 10.2340/actadv.v106.adv-2026-0463 (PMC13370663; doi:10.2340/actadv.v106.adv-2026-0463)
Supplement: Supplementary Material 1. [file ActaDv-106-0463-s0001.pdf]

Supplemental material

Supplementary Table 1. Descriptive statistics for MADRS-S, HADS-A, HADS-D and DLQI scores.

*Abbreviations: MF, Mycosis Fungoides; SS, Sézary Syndrome; MADRS-S, Montgomery-Åsberg Depression Rating Scale – Self-reported; HADS-A, Hospital Anxiety and Depression Scale – Anxiety subscale; HADS-D, Hospital Anxiety and Depression Scale – Depression subscale; DLQI, Dermatology Life Quality Index; IQR, interquartile range; *, data missing for one patient; **, data missing for five patients;***, data missing for 6 patients.*

[a] *Wilcoxon rank-sum test used to calculate p-value, Early MF vs Advanced MF/SS.*

Questionnaire	Total (n=60)	Early MF (n=48)	Advanced MF/SS (n=12)	P-value[a]
**MADRS-S**				
Median (IQR)	7.5 (0.5 – 14)***	7 (1 – 14)**	8 (1.5 – 20.5)*	p = 0.68
Mean (range)	9.5 (0 – 35)***	8.91 (0 – 29)**	11.6 (0 – 35)*	
**HADS-A**				
Median (IQR)	5 (2 – 7)*	4 (2 – 7)*	6 (0.75 – 8)	p = 0.71
Mean (range)	5.12 (0 – 17)*	4.96 (0 – 13)*	5.75 (0 – 17)	
**HADS-D**				
Median (IQR)	2 (1 – 6)*	2 (1 – 4.5)*	3.5 (0.75 – 8.5)	p = 0.52
Mean (range)	3,92 (0 – 16)*	3.51 (0 – 14)*	5.5 (0 – 16)	
**DLQI**				
Median (IQR)	1 (0.5 – 3)*	1 (0.5 – 3)*	1 (0.75 – 7.25)	p = 0.80
Mean (range)	2.8(0 – 13)*	2.64 (0 – 13)*	3.4 (0 – 12)	

Supplementary Table 2. Demographic and clinical characteristics of the MF/SS cohort compared with the psoriasis cohort.

*Abbreviations: MF, Mycosis Fungoides; SS, Sézary Syndrome; NA; not known; SD, standard deviation; BMI, Body Mass Index; *, data missing for two patients; **, data missing for ten patients; ***, data missing for one patient; ****, data missing for 23 patients.*

[a] Fisher's exact test used to calculate p-value, MF/SS vs Psoriasis. Bold font indicates statistically significant p-value.
[b] Wilcoxon rank-sum test used to calculate p-value, MF/SS vs Psoriasis.

Variable	MF and SS (n=60)	Psoriasis (n=139)	P-value
**Sex, n (%)**			
Male	38 (63%)	76 (55%)	
Female	22 (37%)	62 (45%)	$p = 0.35$[a]
NA	0	1	
**Age at diagnosis**			
Median (range)	57.4 (20.0 – 85.6)	54.0 (18.0 – 79.0)*	
			$p = 0.10$[b]
Mean ± SD	56.3 ±17	52.0 ± 17*	
**BMI (kg/m²)**			
Median (range)	26.0 (18.2 – 37.2)**	27.0 (17.3 – 48.0)***	
			$p = 0.06$[b]
Mean ± SD	26.3 ± 4.4**	28.1 ± 6.0***	
**Cardiometabolic disease, n (%)**			
Yes	25 (42%)	91 (78%)****	**$p < 0.001$**[a]
No	35 (58%)	25 (22%)****	
**Depression diagnosis, n (%)**			
Yes	10 (17%)	35 (26%)	
No	50 (83%)	100 (74%)	$p = 0.20$[a]
